# P-418. A Need for Infection Prevention among Patients with ESKD on Maintenance Dialysis: a First Step in Understanding how SARS-CoV-2 Impacts this Population

**DOI:** 10.1093/ofid/ofae631.619

**Published:** 2025-01-29

**Authors:** Shivani Aggarwal, Emily A Hu, Jennifer R Dusendang, Yuval Koren, Cátia Ferreira, Lisa Glasser, Sudhir Venkatesan, Carla Talarico, Della Varghese

**Affiliations:** Landmark Science, Inc., Los Angeles, California; Graticule, Inc., San Francisco, California; Graticule, Inc., San Francisco, California; Graticule,Inc., Newton, Massachusetts; Vaccines and Immune Therapies, BioPharmaceuticals Medical, AstraZeneca, Wilmington, DE, USA, Wilmington, Delaware; Vaccines and Immune Therapies, BioPharmaceuticals Medical, AstraZeneca, Wilmington, DE, USA, Wilmington, Delaware; Medical and Payer Evidence Statistics, BioPharmaceutical Medical, AstraZeneca, Cambridge, UK, Cambridge, England, United Kingdom; Vaccines and Immune Therapies, BioPharmaceuticals Medical, AstraZeneca, Gaithersburg, MD, USA, Gaithersburg, Maryland; AstraZeneca, Gaithersburg, Maryland

## Abstract

**Background:**

Although infections are a main cause of hospitalizations and deaths among patients with end-stage kidney disease (ESKD), contemporary post-pandemic data on the pathogen burden and outcomes among these patients are not well characterized. Identifying patients with ESKD and confirmed maintenance dialysis (MD) from electronic medical records (EMR) is challenging due to fragmented care since the majority ( >85%) receive MD at large dialysis organizations. In this study, patients with ESKD identified from the EMR will be linked to claims to characterize real-world outcomes among patients with confirmed MD by select pathogens to identify novel opportunities for prevention.

**Table.**
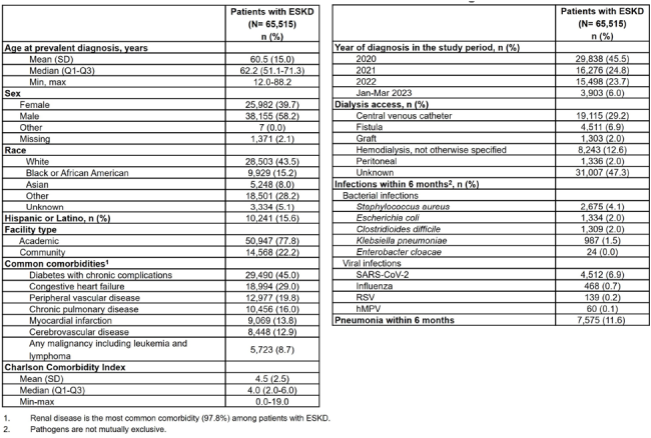
Clinical Characteristics and Infections Among Patients with ESKD between 2020-2023

**Methods:**

As a first step, the infection burden among adolescent and adult patients with a diagnosis of ESKD requiring MD (ICD-10-CM codes N18.6, Z99.2) identified between January 2020-March 2023 within the Loopback Analytics US EMR database was described. Patients were followed for 6 months unless they died or were lost to follow-up. Select infections within 6 months after the ESKD diagnosis date were defined using diagnosis codes or positive laboratory test. Comorbidities, types of vascular access, common pathogens, and proportion of patients with pneumonia were reported.

References

**Figure:**
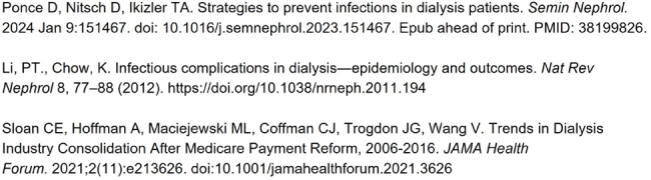

**Results:**

Overall, 65,515 patients with ESKD were identified between 2020-2023 and presented with high comorbidity burden (Table). 29.2% of patients with ESKD had documented central vein catheter, 6.9% had fistulas, 12.6% had hemodialysis without documentation of access type, and 2% had peritoneal dialysis. Follow-up of patients showed *S. aureus* (4.1%), *E. coli* (2.0%), *C. difficile* (2.0%), and *K. pneumoniae* (1.5%) as common bacterial infections. SARS-CoV-2 was the most common viral infection (6.9%). Moreover, 11.6% were diagnosed with pneumonia within 6 months.

**Conclusion:**

The infection burden among patients with ESKD suggests a need for additional preventative strategies in this vulnerable group. EMR data linked to claims is needed to identify patients with ESKD and confirmed MD, and quantify healthcare costs. Evaluation of outcomes such as hospitalization rates, cardiovascular events, and survival among patients with ESKD and confirmed MD by infection type, such as SARS-CoV-2, is ongoing.

**Disclosures:**

**Emily A Hu, PhD, MS**, Graticule, Inc.: Employee|Graticule, Inc.: Stocks/Bonds (Private Company) **Jennifer R Dusendang, MPH**, Graticule, Inc.: Employee|Graticule, Inc.: Stocks/Bonds (Private Company) **Cátia Ferreira, PhD**, AstraZeneca: Employee of AstraZeneca and may hold stock and/or stock options **Lisa Glasser, MD**, AstraZeneca: Employee of AstraZeneca and may hold stock and/or stock options **Sudhir Venkatesan, MPH, PhD**, AstraZeneca: Employee of AstraZeneca and may hold stock and/or stock options **Carla Talarico, PhD, MPH**, AstraZeneca: Employee of AstraZeneca and may hold stock and/or stock options **Della Varghese, PhD, MS, PharmD**, AstraZeneca: Employee of AstraZeneca and may hold stock and/or stock options

